# From the Medical Clinics of Prof. Reigel, at Giessen, for Advancement in the Diagnosis of Acute Miliary Tuberculosis

**Published:** 1884-12

**Authors:** 

**Affiliations:** Lippstringe, formerly Clinical Assistant; Berlin


*From the Medical Clinics of* PROF. REIGEL, *at Giessen, for Advancement in the Diagnosis of Acute Miliary Tuberculosis. By* DR. PROEBSTING, *in Lippstringe, formerly Clinical Assistant.*

Since their discovery, the bacilli of tuberculosis have been sought for and found in the secretions of most tuberculous organs, and through the examination for bacilli in the secretions an immeasurable service has been rendered to diagnosis.

Particularly in the tuberculous diseases of the urine excretory apparatus, the diagnosis was formerly sometimes not, sometimes first made in the later stages of the disease, and uncomplicated renal tuberculosis still remains *inter vitam* incapable of diagnosis.

A number of cases have already appeared in the literature of the profession(1) in which, through the examination of the urine for the bacilli of tuberculosis, a diagnosis of uro-genital tuberculosis has been made. In the cases reported, the question was always concerning a purely local affection.

It has come into my power here, to record a case of renal tuberculosis, with subsequent acute miliary tuberculosis, which in its clinical course contains some points of interest that perhaps may be interesting to all, since here the diagnosis, doubtful as to whether the case was typhoid fever or acute miliary tuberculosis, was first definitely ascertained after an examination for bacilli at a time when all direct symptoms of acute miliary tuberculosis were wanting. For the kindly commission of the history of this case to my care, I am under the

(1) Rosenstein, Centralblatt für die med. Wissenschaften, 1883, No 5. M. Babes, Centralblatt für die med Wissenschaften, 1883, No. 9. Lichtheim Fortschritte der Medicin, 1883, No. 1. Kredel. Sitzungsberichte der med. Gesellschaft zu Giessen, Sitzung vom, 13 Marz, 1883. S. Berichte der oberrh. Ges. if. Natur und Heilkunde Band XVII. R. Singleton Schmidt, Lancet, 1883, No. 1, w. 2.

greatest obligations to my respected former chief, Prof. Riegel.

Katharine Schmidt, twelve years old, first appeared at the clinic July 2d, 1883.

Patient, formerly always healthy, was taken sick February 12th, 1883, on her way to school, with pain and stiffness in her left leg. The pain increased so that she was brought to the surgical clinic towards the end of March. Here the case was diagnosed as *coxitis sinistra*, and an extension apparatus applied.

After the patient had felt for some time entirely well again, she began, on July 1st, to vomit, to have a diarrhœa, and to fever. On this account she was brought to the medical clinic. Her parents had died some years before, cause unknown, and she had one elder sister, living and in good health.

On examination she seemed well nourished. Lungs entirely resonant on percussion. Respiration puerile, with the pulmonary apices entirely free from rales.

Heart normal. Frequency of pulse, 96.

Abdomen sunken. Skin without an exanthem. Epigastrium, on slight pressure, painful. Area of liver dullness, normal. Extremity of the pancreas palpable, and the organ enlarged. Left lower extremity in extension apparatus. *Trochanter major sinister* on pressure, and passive movements of left limb, painful. No œdema. Urine without albumen or sugar. Specific gravity, 1019.

Morning temperature, 37.8° C.; evening temperature, 28.2°C.

July 3d. Three thin fecal discharges. Morning temperature, 37.6°; evening temperature, 39.2°; pulse, 92–96.

July 4th. Diarrhœa has stopped. Morning temperature, 37.8°; evening temperature, 39.1°; pulse, 94–166.

July 6th. Enlargement of the pancreas has increased. No roseola. No fecal discharge. Morning temperature, 37.8°; evening temperature, 38.3°; pulse, 96–104.

July 7th. Morning temperature, 37.6°; evening temperature, 38.5°; pulse, 90–92.

July 10th. Temperature remains as high as on former days, 37.8°; evening temperature, 38,6°. Patient feels pretty well. Pancreas enlargement continues. No roseola. A daily fecal evacuation. Urine not abnormal.

July 12th. Temperature between 28.0° and 38.6°; pulse, 102–104.

July 15th. Temperature since this morning almost normal. Patient feels well. Appetite increases rather slowly. The extended leg causes no complaint.

July 29th. Urine contains a trace of albumen; otherwise *status idem.*

July 23. Subjectively no complaints. Temperature has not increased lately. Appetite very good. Nutritious diet readily digested. Pancreas enlargement remains unaltered. Urine again without albumen.

July 29th. Pancreas still enlarged as before. Strength of patient moderate. Recuperation very slow.

July 30th. To-day, for the first time, a rise again in the evening temperature to 38.2° C. Subjectively, vomiting; considerable feebleness; no complaint.

Aug. 3d. Morning temperature during last three days, 37.2°; evening temperature up to 38.2°; pulse, 96–112. Lungs normal. No cough. No diarrhœa. No roseola. Pancreas, as before, considerably enlarged. Urine normal.

Aug. 6th. Evening temperature continually higher. Urine again shows, on examination with ferro-cyanide of potassium and vinegar, a well marked cloudiness.

Aug. 8th. Temperature falls slowly to the normal; this evening, 37.4°; pulse, 102. Pancreas enlargement unchanged. Urine, yesterday and to-day, without albumen. Increased feebleness and emaciation. At the *os sacrum*, beginning decubitus (bed sores).

Aug. 9th. Urine exhibited again albumen; microscopically, its sediment shows the presence of epithelial cells, lymph corpuscles and detached hyaline casts.

Aug. 11. *Status idem.* Urine unchanged. On examination the sediment shows positively the presence of the bacilli of tuberculosis. In almost every specimen, groups of from two to six in a group are found.

Aug. 14th. Temperature normal continually. Health, in general, poor. Strength becomes more and more feeble. Less appetite. *Decubitus* at the *os sacrum* unchanged. Subjectively no complaints. No headache. No diarrhœa.

Aug. 16th. A little vomiting. A thin fecal evacuation. Patient very apathetic and non-complaining; only passive movements of the left limb cause expressions of pain. Pancreas enlargement and urine as before. On ophthalmoscopic examination a few yellow spots are observed in the retina.

August 17. Complete stupor. Feces and urine are voided involuntarily. Temperature, till to-day normal, rises to 38.2° Pulse strong, 92 beats. Respiration regular.

Aug. 19th. Unbroken somnolence. Proneness to vomiting. Convulsions. Cheyne-Stokes' respiration. Pulse slow but still full. Temperature 37.8°–38.3°.

Aug. 22d. After an increase of these symptoms, *exitus letalis* occurred to-day.

Post-mortem report of Aug. 23d (Dr. Wesener):

Corpse very much emaciated, with a greenish discolored abdomen, which is drawn in. Left lower extremity ca. 1 ctm. (one-third inch) shorter than the right. The epidermis of the left lower extremity very dry, brownish discolored, and has large brownish bands.

Skull short and broad. Frontal suture still existing. Grooves for the blood-vessels very deep. *Dura* somewhat firm, not very well supplied with blood. *Pia*, firm (in its convex position), and soaked in a yellowish fluid, especially so at the *sulci.* On removing the brain a large quantity of yellow serous fluid was found. At the base the *pia* still richer in blood; moreover, at the floor of the third ventricle and over the *pons varolii*, it is steeped in a briny and turbid liquid. At the sinuses, likewise on the inferior surface of the cerebellum, were found a large number of grayish-yellow miliary tubercles. Both lateral ventricles were wide and contained a considerable quantity of turbid serous fluid. Brain substance moist, pulpy, well supplied with blood, only the gray matter somewhat pale. The third and fourth ventricles seemed similar to the lateral ventricles.

The diaphragm on the right side, at the lower border of the fourth rib; on the left side, at the upper border of the fourth rib.

After opening the thoracic cavity, the left lung sank back somewhat, and seemed only bound down to the walls of the thorax by slight adhesions. The right, on the other hand, is strongly adherent. Almost the whole pericardium lies exposed. It contains a small quantity of clear, yellowish serum.

Heart, strongly contracted, contained in both auricles, especially in the right one, a number of clots composed of red and white blood corpuscles in meshes of fibrin. Heart muscle pretty firm in texture, brownish-yellow. Valves intact.

Left lung, adherent in several places, together with the pleura covering it, presented considerable number of grayish red nodules. On making a section through it, it was found pretty compact, pigmented, and to contain air. The upper lobe pale, the lower one somewhat richer in blood, and scattered through each a large number of gray nodules.

Right lung firmly adherent to the thoracic wall and the diaphragm; on exposing it, a few grayish nodules were discernible. On making a section the superior lobe, light red in color, with a number of paler spaces in different parts, contained air in its cells, and a large number of grayish-red nodules. Middle lobe similar to the upper one. The inferior lobe appeared tougher in structure, darker, contained less air in its cells, somewhat compressed, and contained, likewise, a large number of miliary nodules. Mucous membrane of the bronchi, reddish. Both lungs contained a large quantity of a frothy liquid.

Papillæ at the base of the tongue very prominent. Right tonsil somewhat enlarged; mucous membrane of the larynx and trachea without any other peculiarity.

Pancreas in close contact with the diaphragm, somewhat large, 12 ctm. long, 7 ctm. broad (five by three and one-third inches). On making a section through it, the follicles were pretty plain between them, as upon the capsule were scattered yellowish nodules. The stomach contained a greenish fluid; its mucous membrane was pale, without further peculiarity.

Liver, adherent to the anterior abdominal wall and diaphragm, presented upon its capsule a number of whitish nodules. On making a section through it, it appeared yellowish-gray-red in color, with the lobules not well marked, flabby in consistency, and interspersed in it were a large number of whitish and yellowish nodules.

Gall bladder was filled with dark bile.

Bladder was also well filled and adherent anteriorly and laterally to the pelvis.

The kidneys were removed, together with the reproductive organs and bladder. Mucous membrane of the bladder, at the urethral opening, strongly spotted, dark reddish in color, and contained a large number of whitish nodules.

Uterus about the size of that of a girl three to four years old; showed otherwise nothing of interest. Ovaries without any peculiarity.

Right kidney flabby; showed on its anterior surface injected *stellulæ* and a number of whitish points. On making a section through it, the cortical substance appeared pale and yellowish-red.

Medullary substance dark, especially at the borders of the pyramids; besides this, there are a large number of nodules of a caseous character in the cortical and medullary portions. In the left kidney these changes are more strongly pronounced. Here a number of pyramids have already undergone caseous degeneration.

The internal surfaces of the uterus show no alteration.

Mucous membrane of the small intestines, pale, in its lower portion only more reddish. Close to the valve (ileo-cæcal) a caseous follicle, and also a thickened blackish spot containing small whitish nodules.

In the *process. vermif.* an *ascaris.* Mesenteric glands unaltered.

On opening the acetabular cavity, a thick greenish-yellow pus was discharged. The cavity itself was in an advanced stage of destruction. The cartilage had been destroyed in two places; in one, the cavity formed was about the size of a cherry pit; in the other, the size of a five cent piece, so that the acetabulum looked like a dark grayish, discolored mass of bone. The cartilage remaining was yellow opaque, and could readily be peeled off and broken. The head of the femur was still covered with cartilage, though it fitted loosely in the cavity, and had already assumed a necrotic appearance. The spongy substance of the head and neck was dark red in color, very soft and frangible. Just below its cartilage there was a caseous mass about the size of a cherry pit.

In the choroid coat of the left eye, posteriorly, were found a number of yellowish-white nodules.

At the thoracic duct, nothing of especial interest.

Anatomical diagnosis: *Tuberculosis miliaris acutis.* Coxitis tuberc. sin. Tuberculosis renum et vesicæ.(2) *Tubercula disseminata pulmonum. Meningitis acuta tuberculosa. Hydrocephalus internus. Tubercula lienis et hepatis et chorodicæ sin.*

This case presented for diagnosis a number of difficulties. In the beginning it occurred to me that the case was a light form of typhoid, towards which the gastric symptoms, the pancreas enlargement and the fever, though irregular, pointed. But, as after the cessation of the fever, the patient did not begin to recuperate, this seemed more and more improbable, since the pancreas enlargement also remained. The fever also recurring at a later stage could not be referred to a relapsing typhoid.

So the presumption that the case was one of miliary tuberculosis became stronger; the more so, as likewise the suspicion that we were dealing with a tubercular inflammation in the left trochanteric region, did not seem unfounded; yet, for the diagnosis of acute miliary tuberculosis, every positive symptom was wanting. The whole course of the disease showed

---

(2) Bezuglich des mikroskopishen Befundes dieses Falles sei verweisen auf Wesener; Ueber das vorkommen der Tuberkelbacillen in den Organen Tuberkulöser. Deutches Archiv. fur Klin. Med. Band 34. Heft 5, w. 6. S. 707. Fall 25.

that it was not an ordinary case of acute miliary tuberculosis. Then came the irregular appearance in the urine, of albumen, together with a considerable quantity of epithelial and lymph cells in the sediment. This led to the examination of the sediment for the bacilli of tuberculosis, through which we obtained a positive result. Hence it was established that we had a tuberculous disease of the kidneys, and this fact, together with the entire course of the disease, allowed us to suppose that we probably had a general tuberculosis. It was presumed that the starting point of the acute tuberculosis was in the diseased left trochanteric region, and that the disease spread thence to the kidneys and other organs. The post-mortem has proven this supposition to have been correct. Moreover, the causation of all the clinical symptoms, the fever, the pancreas enlargement, diarrhœa, emaciation, *et cetera*, became plain, and thus were established at once both diagnosis and prognosis at a time when no direct sign of acute miliary tuberculosis was prominent, and when the ophthalmoscopic examination also was of a negative result. First, later on, did the typical signs of the disease appear, and then was it possible to determine the presence of tubercular nodules on the left choroid.

This case, of such importance to diagnosis, was rendered possible to diagnosticate through the fact that the kidneys were strongly affected and had partly undergone caseous degeneration. In other cases, even where the miliary tubercles are generally disturbed, often only a few nodules can be found in the kidneys, which *intra vitam* produce no symptoms.

These miliary tubercles which, according to the investigations of Arnold(3) establish themselves by preference in the remote parenchyma of the straight and convoluted tubes, lead first, at a later stage, to caseous degeneration and to the excre-

(3) Arnold. Ueber Nierentuberkulose. Virch. Arch. Bd. 83. Heft. 2.

tion of tuberculous material through the urinary passages. It will only be possible in exceptional cases to make the examination for bacilli in the urinary sediment a useful diagnostic aid, as in like manner the examination of the sputa in this disease, according to Demme(4) d'Espine(5) and others, is generally of little avail.

*Note.*—The temperature quoted above is the axillary temperature according to Celsius. To get the Fahr. temp. multiply the degrees by $\frac{9}{5}$ and add 32.

From the Berliner Klin. Wochenschrift, No. 37, 1884. Translated by E. A. Boas, M.D.

Berlin, Oct. 11th, 1884.

---

(4) Demme. Berliner Klin. Wochenschift, 1883, No. 15.

(5) d'Espine Revue Med. d. 1. Suisse Komande, 1882, No. 12. (Mit. von Ziehl. Deutche Med. Wochenschrift, 1883, No. 5).

